# Supplementary material for: Are 150 km of open sea enough? Gene flow and population differentiation in a bat-pollinated columnar cactus
Source: PLoS One. 2023 Jun 29;18(6):e0282932. doi: 10.1371/journal.pone.0282932 (PMC10309638; doi:10.1371/journal.pone.0282932)
Supplement: S1 Table — Name, geographical location, elevation, and vegetation type of Stenocereus thurberi populations used in this study. The peninsular group are populations in Baja California Sur (México) and the mainland group are populations in Sonora (México). (DOCX) [file pone.0282932.s001.docx]

| **Population** | **Latitude N** | **Longitude W** | **Elevation [m]** | **Vegetation** |
| --- | --- | --- | --- | --- |
| **Mainland Group** | |  |  |  |
| Carbó [Cr] | 29° 34´ 06´´ | 111° 05´29´´ | 501 | Plains of Sonora^1^ |
| Las Guásimas [Gs] | 27° 52´ 10´´ | 110° 28´ 55´´ | 5 | Central Gulf Coast^1,2^ |
| Kino [Kn] | 28° 52´ 56´´ | 112° 01´ 39´´ | 60 | Central Gulf Coast^1^ |
| Magdalena [Mag] | 30° 40´ 57´´ | 110° 58´ 46´´ | 964 | Arizona Upland^1^ |
| Sonoyta [Son] | 31° 48´ 17´´ | 112° 51´ 56´´ | 512 | Arizona Upland^1^ |
| Tecoripa [Tec] | 28° 37´ 50´´ | 109° 57´ 42´´ | 411 | Foothills Thornscrub^2^ |
| Zacate Blanco [ZB] | 26° 38´ 12´´ | 109° 18´ 39´´ | 15 | Coastal Thornscrub^2^ |
| **Peninsular Group** |  |  |  |  |
| Balandra [Bal] | 24° 19´ 22´´ | 110° 18´ 45´´ | 11 | Central Gulf Coast^1^ |
| El Palmar [Pal] | 23° 16´ 24´´ | 110° 08´ 11´´ | 58 | Coastal Thornscrub^2^ |
| Nopolo [No] | 25° 56´ 06´´ | 111° 22´ 11´´ | 63 | Central Gulf Coast^1^ |
| Santa Rosalía [SRo] | 27° 22´ 50´´ | 112° 23´ 31´´ | 372 | Central Gulf Coast^1^ |
| San Francisco [SFr] | 27° 28´ 15´´ | 113° 13´ 19´´ | 182 | Vizcaíno Region^1^ |

^1^Sonoran Desert subdivision [Shreve F. Vegetation of the Sonoran Desert. Washington: Carnegie Institution of Washington; 1951]. ^2^Transition from Sonoran Desert to thornscrub. See Búrquez et al. [81].
