# Supplementary material for: Are 150 km of open sea enough? Gene flow and population differentiation in a bat-pollinated columnar cactus
Source: PLoS One. 2023 Jun 29;18(6):e0282932. doi: 10.1371/journal.pone.0282932 (PMC10309638; doi:10.1371/journal.pone.0282932)
Supplement: S2 Table — The PC1 was most affected by precipitation bioclimatic variables, while seasonal precipitation and annual temperature had the most influence on the PC2. For PC3, the maximum temperature of the warmest month, seasonality, and isothermality contributed with significant variance. (DOCX) [file pone.0282932.s002.docx]

| **PC1 33.9%** | |
| --- | --- |
| *Variable* | *R2* |
| BIO2 mean diurnal range | 0.54 |
| BIO6 min temperature of coldest month | 0.62 |
| BIO12 annual precipitation | 0.68 |
| BIO17 precipitation of driest quarter | 0.87 |
| **PC2 33.4%** | |
| *Variable* | *R2* |
| BIO1 annual mean temperature | 0.60 |
| BIO15 precipitation seasonality | 0.73 |
| **PC3 13.8%** | |
| *Variable* | *R2* |
| BIO3 isothermality | 0.64 |
| BIO4 temperature seasonality | 0.68 |
| BIO5 max temperature of warmest month | 0.67 |
