# Supplementary material for: Are 150 km of open sea enough? Gene flow and population differentiation in a bat-pollinated columnar cactus
Source: PLoS One. 2023 Jun 29;18(6):e0282932. doi: 10.1371/journal.pone.0282932 (PMC10309638; doi:10.1371/journal.pone.0282932)
Supplement: S3 Table — Pink shade, absent from that group. Blue shade, most common within a group. Green shading, unique haplotypes. (DOCX) [file pone.0282932.s003.docx]

| **POPULATION** | **HAPLOTYPES** | | | | | | | | | | | |  |
| --- | --- | --- | --- | --- | --- | --- | --- | --- | --- | --- | --- | --- | --- |
|  | **H1** | **H2** | **H3** | **H4** | **H5** | **H6** | **H7** | **H8** | **H9** | **H10** | **H11** | **H12** | **Total** |
| ***Mainland Group*** |  |  |  |  |  |  |  |  |  |  |  |  |  |
| *Carbó* | x | 0.308 | x | 0.231 | 0.308 | 0.154 | x | x | x | x | x | x | 13 |
| *Guásimas* | x | 0.429 | x | 0.429 | x | x | 0.143 | x | x | x | x | x | 14 |
| *Kino* | x | 0.357 | x | 0.357 | x | x | x | 0.286 | x | x | x | x | 14 |
| *Magdalena* | x | 0.125 | x | 0.250 | 0.313 | 0.313 | x | x | x | x | x | x | 16 |
| *Sonoyta* | x | x | x | 0.412 | x | x | x | x | x | 0.588 | x | x | 17 |
| *Tecoripa* | x | 0.267 | x | 0.333 | x | x | x | x | x | x | 0.400 | x | 15 |
| *Zacate Blanco* | x | 0.333 | x | 0.267 | x | x | x | x | x | x | 0.067 | 0.333 | 15 |
| ***Total mainland*** | 0 | 0.250 | 0 | 0.327 | 0.087 | 0.067 | 0.019 | 0.038 | 0 | 0.096 | 0.067 | 0.048 | 104 |
| ***Peninsular group*** |  |  |  |  |  |  |  |  |  |  |  |  |  |
| *Balandra* | 0.500 | 0.143 | 0.357 | x | x | x | x | x | x | x | x | x | 14 |
| *Nopolo* | x | x | x | x | x | 0.154 | 0.538 | 0.308 | x | x | x | x | 13 |
| *El Palmar* | 0.400 | x | x | x | x | x | x | 0.267 | 0.333 | x | x | x | 15 |
| *San Francisco* | x | x | 0.071 | 0.143 | x | x | x | 0.786 | x | x | x | x | 14 |
| *Santa Rosalía* | x | x | x | x | x | x | x | 1.000 | x | x | x | x | 16 |
| ***Total peninsular*** | 0.181 | 0.028 | 0.083 | 0.028 | 0 | 0.028 | 0.097 | 0.486 | 0.069 | 0 | 0 | 0 | 72 |
| **Total** | 0.074 | 0.159 | 0.034 | 0.205 | 0.051 | 0.051 | 0.051 | 0.222 | 0.028 | 0.057 | 0.040 | 0.028 | 176 |
