# Supplementary material for: Are 150 km of open sea enough? Gene flow and population differentiation in a bat-pollinated columnar cactus
Source: PLoS One. 2023 Jun 29;18(6):e0282932. doi: 10.1371/journal.pone.0282932 (PMC10309638; doi:10.1371/journal.pone.0282932)
Supplement: S4 Table — The AMOVA was performed with three molecular markers of chloroplast. (DOCX) [file pone.0282932.s004.docx]

| **Source of variation** | **df** | **Sum of**  **Squares** | **Variance**  **component** | **Percentage of**  **variation** | **Fixation**  **indices** |
| --- | --- | --- | --- | --- | --- |
| Among geographic groups | 1 | 64.123 | 0.736 | 44.38 | *F*_CT_ =0.443** |
| Among populations within  geographic groups | 10 | 52.404 | 0.339 | 20.46 | *F*_SC_=0.367** |
| Within populations | 164 | 89.219 | 0.583 | 35.16 | *F*_ST_=0.648** |
| Total | 174 | 205.745 | 1.658 |  |  |

df = degree of freedom; ***P* < 0.05
