# Supplementary material for: Are 150 km of open sea enough? Gene flow and population differentiation in a bat-pollinated columnar cactus
Source: PLoS One. 2023 Jun 29;18(6):e0282932. doi: 10.1371/journal.pone.0282932 (PMC10309638; doi:10.1371/journal.pone.0282932)
Supplement: S5 Table — Below the diagonal, distances in km, above the diagonal, genetic differentiation [pairwise Fst]. (DOCX) [file pone.0282932.s005.docx]

|  | *Balandra* | *Carbó* | *Guásimas* | *Kino* | *Magdalena* | *Nopolo* | *El Palmar* | *San*  *Francisco* | *Santa*  *Rosalía* | *Sonoyta* | *Tecoripa* | *Zacate*  *Blanco* |
| --- | --- | --- | --- | --- | --- | --- | --- | --- | --- | --- | --- | --- |
| *Balandra* | - | 0.62 | 0.68 | 0.55 | 0.61 | 0.41 | 0.02 | 0.57 | 0.43 | 0.77 | 0.71 | 0.67 |
| *Carbó* | 602.5 | - | 0.18 | 0.14 | 0.07 | 0.25 | 0.6 | 0.82 | 0.6 | 0.58 | 0.39 | 0.3 |
| *Guásimas* | 406.1 | 227 | - | 0.11 | 0.17 | 0.25 | 0.66 | 0.86 | 0.65 | 0.42 | 0.18 | 0.16 |
| *Kino* | 543.2 | 119 | 288 | - | 0.18 | 0.13 | 0.55 | 0.67 | 0.39 | 0.49 | 0.29 | 0.22 |
| *Magdalena* | 709.7 | 299.4 | 227 | 217 | - | 0.22 | 0.58 | 0.77 | 0.59 | 0.56 | 0.38 | 0.31 |
| *Nopolo* | 202.3 | 401.8 | 230 | 328 | 523.8 | - | 0.37 | 0.54 | 0.32 | 0.51 | 0.38 | 0.34 |
| *El Palmar* | 117.5 | 705.7 | 510.4 | 651 | 829.2 | 323.5 | - | 0.66 | 0.52 | 0.77 | 0.7 | 0.66 |
| *San*  *Francisco* | 457.8 | 316.2 | 274.5 | 199 | 423.3 | 251.6 | 561.5 | - | 0.07 | 0.92 | 0.87 | 0.78 |
| *Santa*  *Rosalía* | 402.4 | 277.2 | 196.4 | 175 | 394.2 | 191.3 | 509.4 | 84.3 | - | 0.78 | 0.69 | 0.62 |
| *Sonoyta* | 873.4 | 299.4 | 493 | 333 | 222.1 | 662.9 | 981.6 | 486.9 | 494.5 | - | 0.42 | 0.39 |
| *Tecoripa* | 492.6 | 240 | 99 | 203 | 240 | 327.9 | 593.4 | 346.9 | 278.9 | 448 | - | 0.036 |
| *Zacate*  *Blanco* | 285.4 | 368 | 178 | 364 | 470 | 226.8 | 379.8 | 401.9 | 314.5 | 668 | 230 | - |
