# Supplementary material for: Are 150 km of open sea enough? Gene flow and population differentiation in a bat-pollinated columnar cactus
Source: PLoS One. 2023 Jun 29;18(6):e0282932. doi: 10.1371/journal.pone.0282932 (PMC10309638; doi:10.1371/journal.pone.0282932)
Supplement: S6 Table — The multiple regressions were used to find the most influential variables on determining the ecological niche model for Stenocereus thurberi. Subscript all = all populations, n = 12, P = only peninsular, N = 5, C = only mainland, n = 7. (DOCX) [file pone.0282932.s006.docx]

| **Equation Linear Model y = b_0_ + b_1_x_1_ + b_2_x_2_ ...** | ***R^2^_adj_*** | ***P*- value** |
| --- | --- | --- |
| π_all_= 0.0089 · pp seasonality - 0.0728 · mean temp + 1.2349 | 0.528 | 0.05 |
| *Hd*_all_ = 0.00079 · annual pp + 0.36 | 0.295 | 0.039 |
| PENINSULAR |  |  |
| π_P_ = 0.00495 · annual pp - 0.3499 | 0.856 | 0.016 |
| *Hd*_P_ = 0.003924·annual pp - 0.09713 · mean temp + 1.8816 | 0.983 | 0.028 |
| CONTINENTAL |  |  |
| π_C_ Not Significant | - | >0.1 |
| *Hd*_C_ = 0.01246·isotherm - 0.01759 · mean temp + 0.41175 | 0.861 | 0.039 |
| **Annual precipitation Inverse Model y = b_0_ + [b_1_ · X^-1^]** | ***R^2^_adj_*** | ***P*- value** |
| π_all_ = 0.742 + [-48.3431 · annual pp^-1^] | 0.265 | 0.087 |
| *Hd*_all_ = 0.918 + [-72.599 · annual pp^-1^] | 0.808 | 0.0001 |
| PENINSULAR |  |  |
| π_P_ = 1.341 + [-124.051 · annual pp^-1^] | 0.951 | 0.005 |
| *Hd*_P_ = 1.072 + [-796.327 · annual pp^-1^] | 0.933 | 0.008 |
| CONTINENTAL |  |  |
| π_C_ Not Significant | - | >0.1 |
| *Hd*_C_ Not Significant | - | >0.1 |
