# Supplementary figures and images for: Are 150 km of open sea enough? Gene flow and population differentiation in a bat-pollinated columnar cactus
Source: PLoS One. 2023 Jun 29;18(6):e0282932. doi: 10.1371/journal.pone.0282932 (PMC10309638; doi:10.1371/journal.pone.0282932)

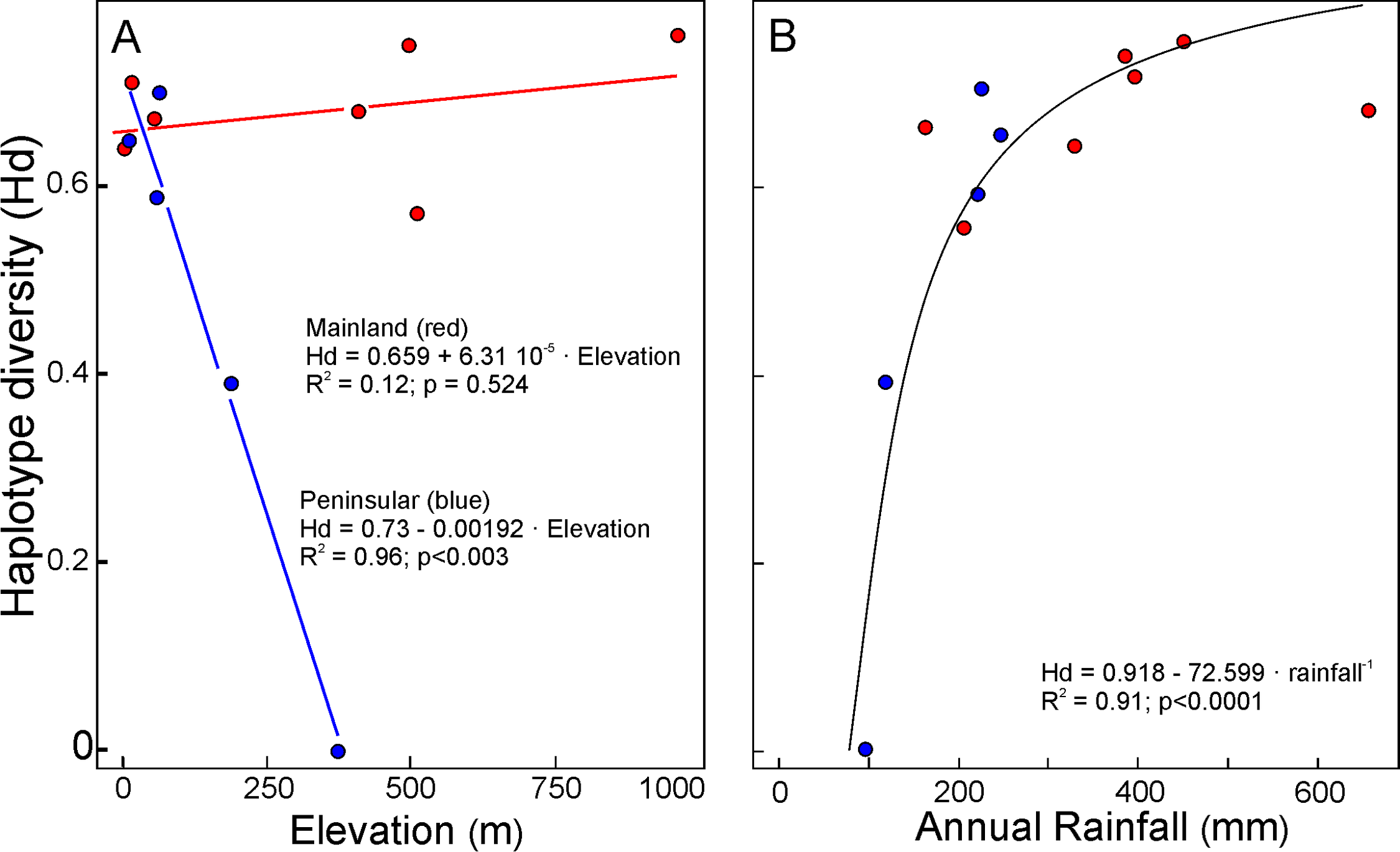

Supplement: S1 Fig — Red = mainland populations, Blue = peninsular populations of Stenocereus thurberi. (TIF) [file pone.0282932.s007.tif]

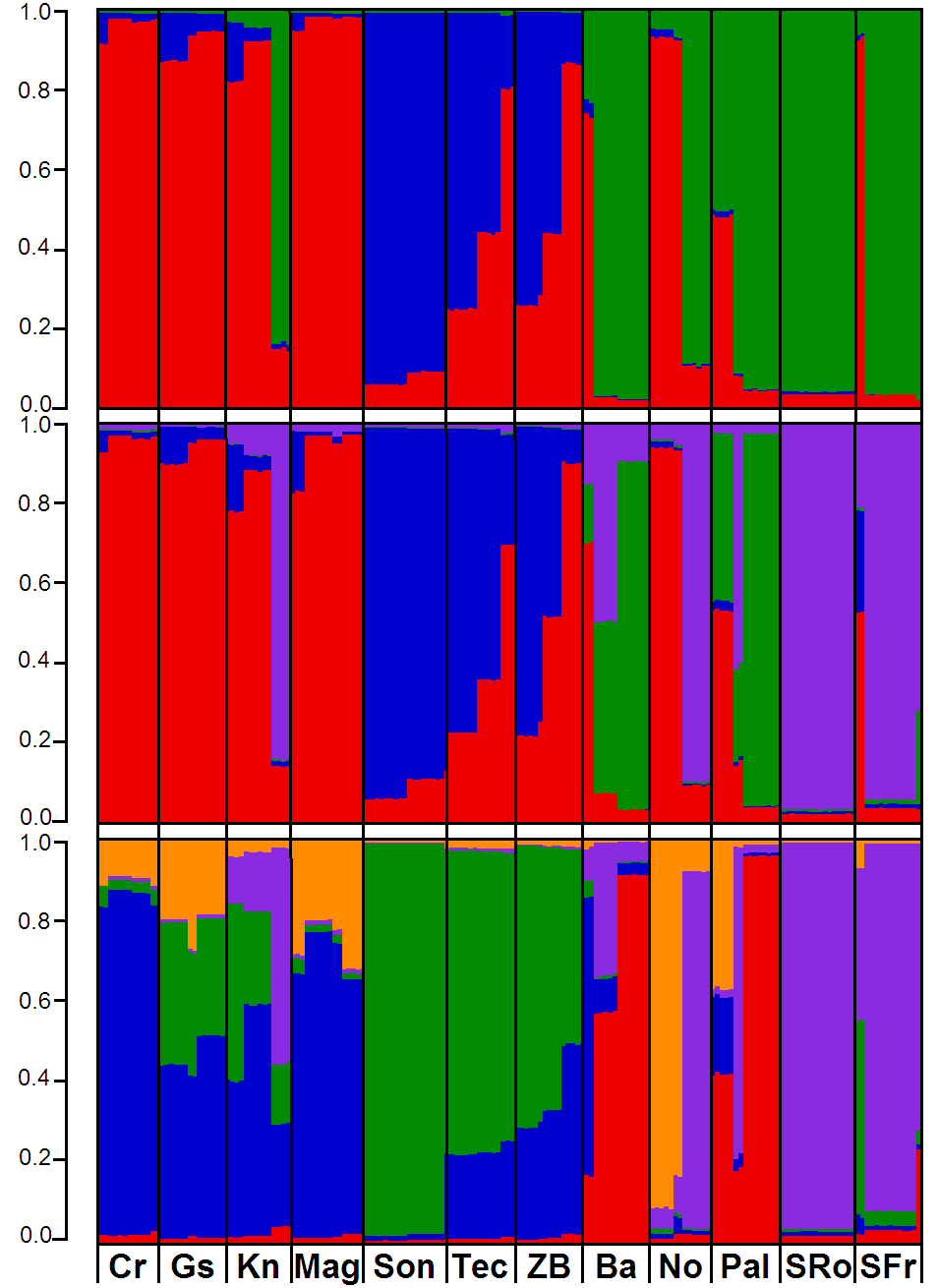

Supplement: S2 Fig — The individual proportion of membership shown for three, four and five clusters [from top to bottom]. The codes of the populations are the same as those in S1 Table. (TIF) [file pone.0282932.s008.tif]

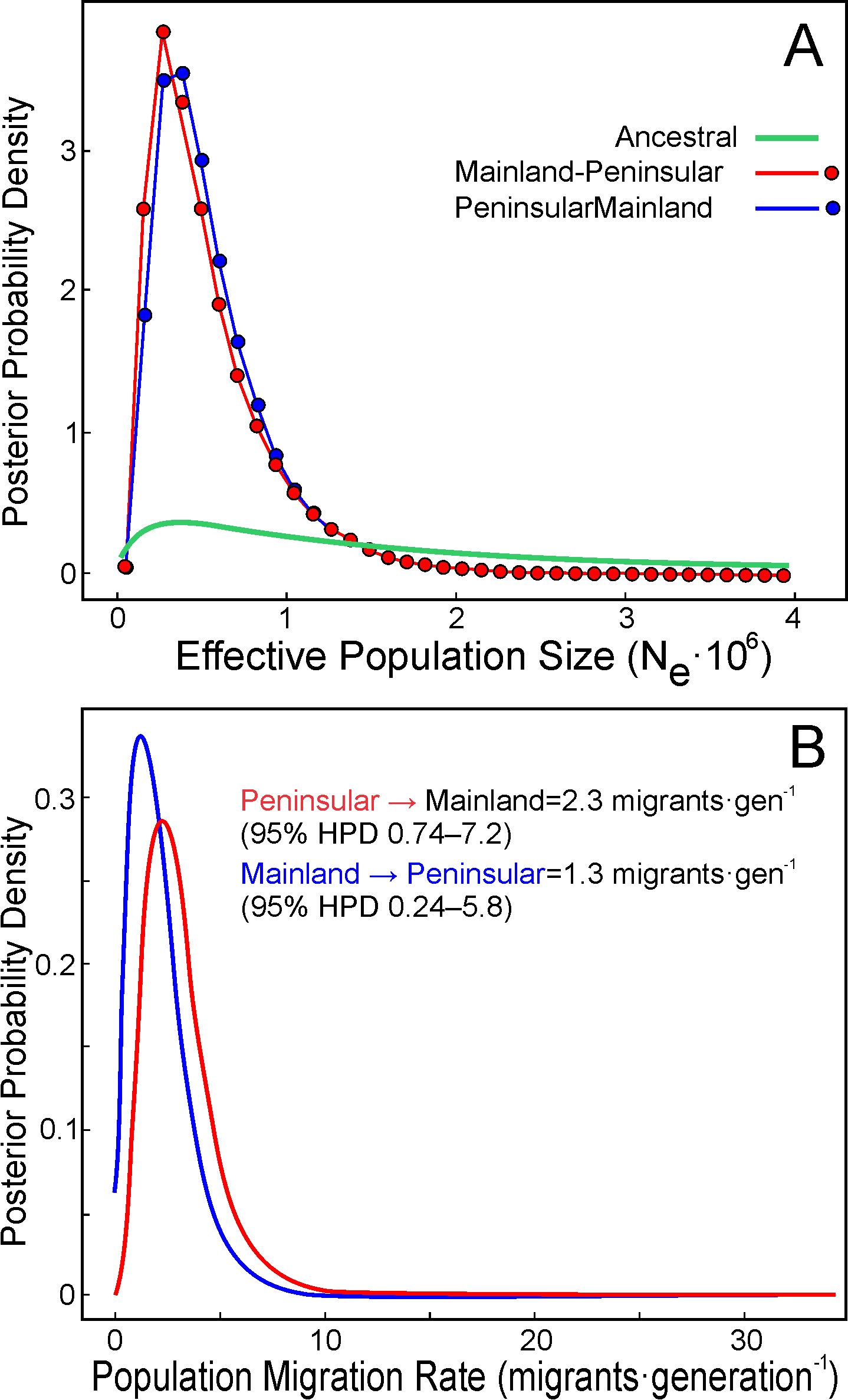

Supplement: S3 Fig — Demographic estimates were calculated by the isolation with migration model of Ossowski et al. [57] mutation rate [7 × 10^−9^ mutations per site per year], and 20 y generation time. A) Estimates of the effective population size for ancestral, mainland, and peninsular populations. B) Population migration rates, mainland → peninsula [blue] and peninsula → mainland [red]. HPD = Highest Posterior Density. (TIF) [file pone.0282932.s009.tif]

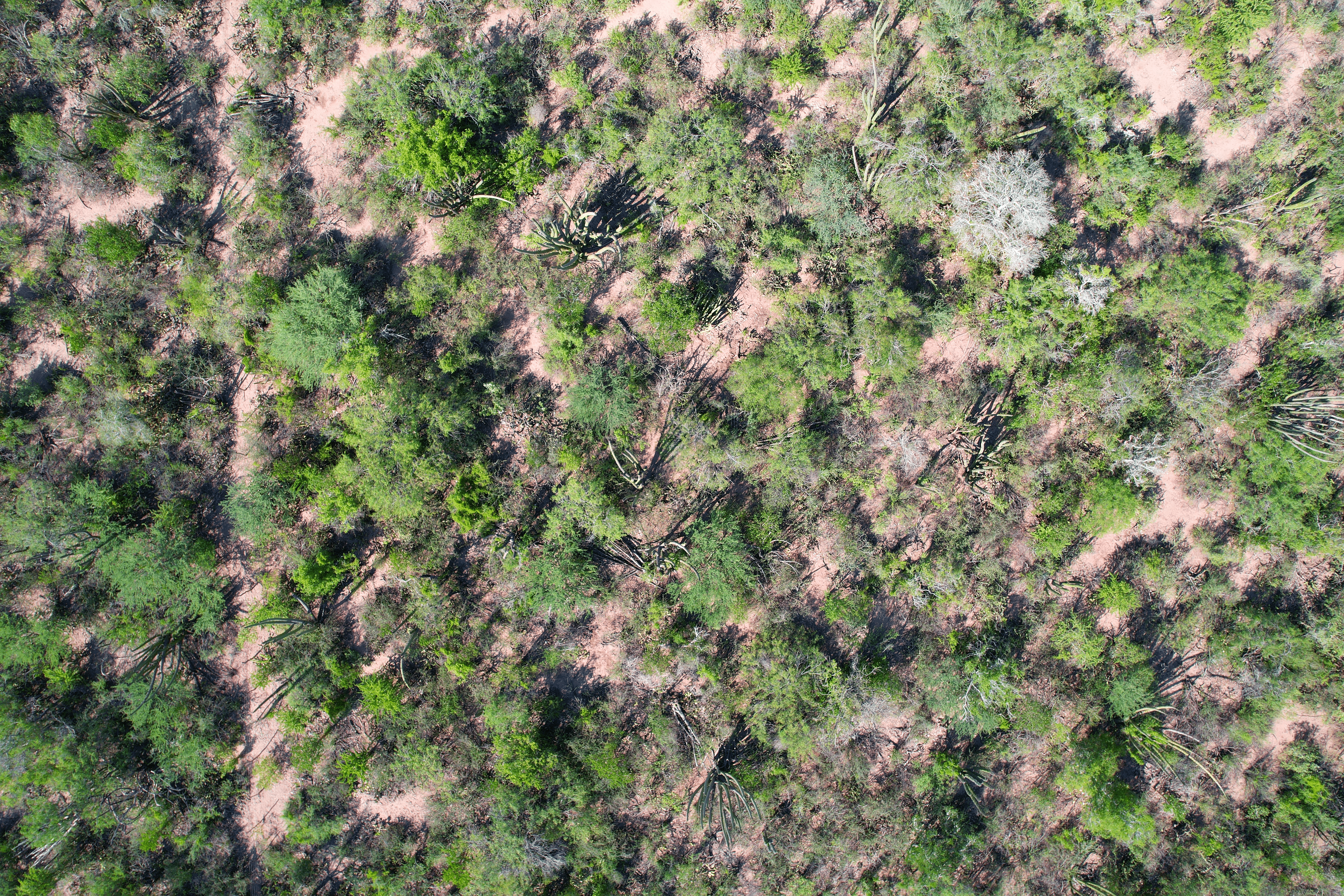

Supplement: S1 Striking image — (GIF) [file pone.0282932.s011.gif]

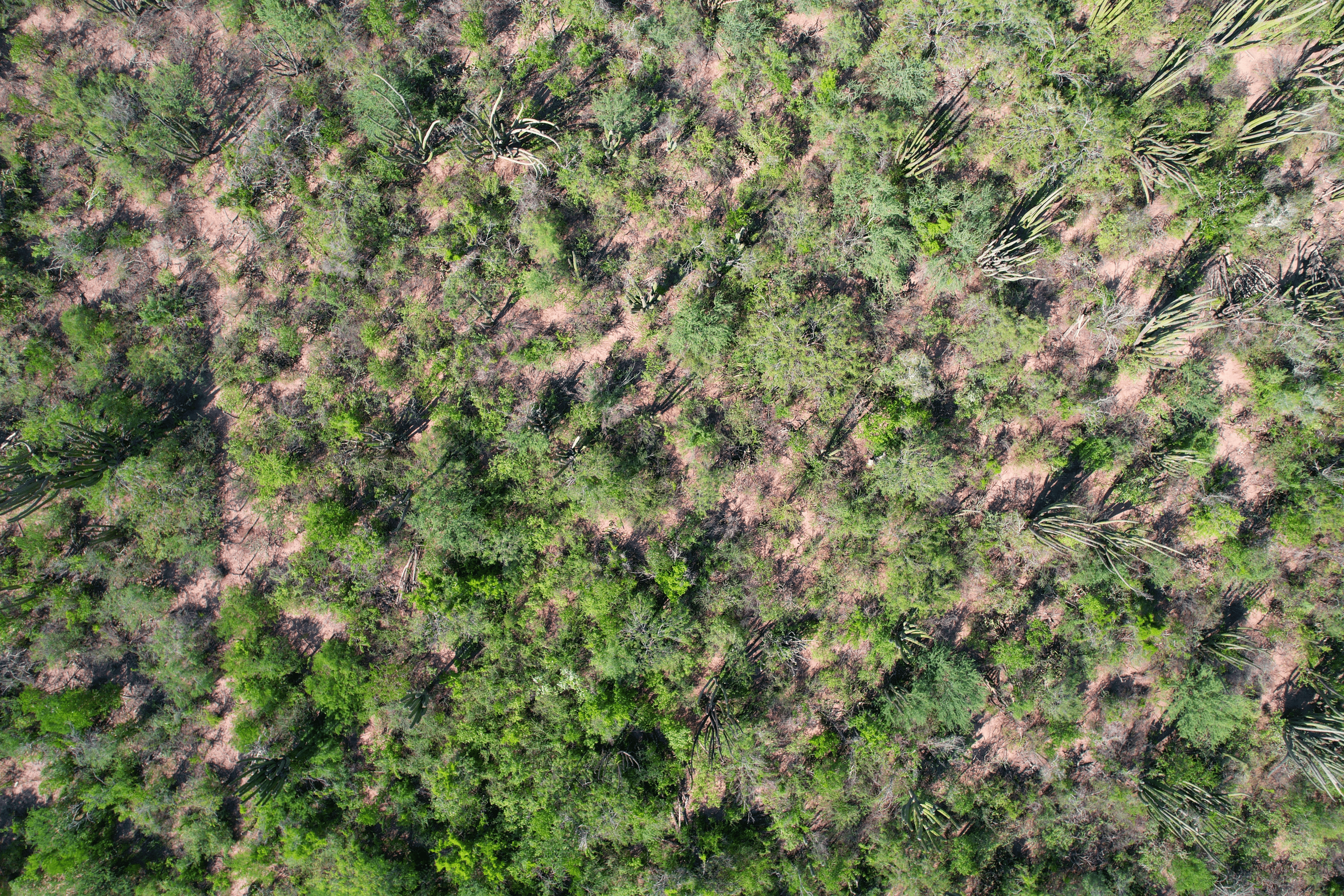

Supplement: S2 Striking image — (GIF) [file pone.0282932.s012.gif]
