# Supplementary material for: Are 150 km of open sea enough? Gene flow and population differentiation in a bat-pollinated columnar cactus
Source: PLoS One. 2023 Jun 29;18(6):e0282932. doi: 10.1371/journal.pone.0282932 (PMC10309638; doi:10.1371/journal.pone.0282932)
Supplement: S1 Methods — (DOCX) [file pone.0282932.s010.docx]

**PCR Amplifications**

PCR amplifications were conducted in a GeneAmp® PCR system 2700 (Applied Biosystems) using 25 µL of total reaction master mix, containing a mixture of 1X buffer reaction (100 mM Tris-HCL, 500 mM KCl, 10μg/ml gelatin, 1%), 1 mM MgCl_2_, 200 µmol/L dNTPs, 0.3 µmol/L of each primer, and 1 U of Taq polymerase, and 25-50 ng of total DNA. The PCR cycles: an initial denaturation at 94°C for 5 min; followed by 35 cycles at 94°C during 0.5 min, 56°C for 0.5 min, and 72°C for 1 min; with a final process of extension of 72°C for 8 min, and we stored the amplification products at 4°C.

The PCR products were purified and sequenced at the High-Throughput Genomics Unit at Washington University, Seattle, USA, using the Sanger method. Chromatogram traces were checked by eye and manually edited if needed with BioEdit v5.0.6 [1]. An assembly of the forward and reverse reads was done using the Phrap-Phred, Consed v.19.0 software [2, 3].

**Parameters of Bayesian inference**

Two simultaneous runs with four Markov chains each were run for 2 × 10^7^ generations and sampled every 500 generations. The first 25% of generations were discarded as burn-in. Convergence of runs was assessed by examination of the average standard deviation of split frequencies and the potential scale reduction factor [4]. In addition, stationarity was confirmed by examining posterior probability, log likelihood, and all model parameters by the effective sample sizes (ESSs) in the program Tracer v1.6 [5].

**Genetic Structure**

Twenty independent runs of 10,000,000 Markov chain Monte Carlo repetitions after a burn-in period of 100,000 iterations were performed in two independent types of searches using the admixture model with correlated allelic frequencies without prior information. For the first type of search, we conducted the STRUCTURE analysis for the whole sample forcing a *K* = 2 (gene pools) to identify mixture between groups. We also performed independent runs for *K* - values ranging from one to ten. The second type of search was carried out using each independent group; the optimal number of gene pools (*K*) was determined by varying the *K* value from 1 to 8 and executing the analysis 20 times per *K* value to determine the maximum value posterior likelihood (lnP (*K*)) [6]. We selected the most probable *K* value using the maximum value of Δ*K* according to Evanno et al. [7]. Additionally, we performed a principal component analysis (PCA) to identify differences in the genetic structure within the groups described using the adegenet v1.4-2 package in R [8].

**References**

1. Hall P, BioEdit. 2001. Version 5.0.6, Department of Microbiology, North Carolina State University, Raleigh.

2. Ewing B, Hillier L, Wendl M, Green P. 1998. Base calling of automated sequencer traces using phred. I. Accuracy assessment. Genome Res. 8: 175-185.

3. Gordon D, Abajan C, Green P. 1998. Consed: a graphical tool for sequence finishing. Genome Res. 8: 195-202.

4. Beiko RG, Keith JM, Harlow TJ, Ragan MA. 2006. Searching for convergence in phylogenetic Markov chain Monte Carlo. Syst Biol. 55: 553–565.

5. Rambaut A, Drummond AJ, Xie D, Baele G Suchard MA. 2008. Posterior summarization in Bayesian phylogenetics using Tracer 1.7. Syst Biol 67: 901–904.

6. Pritchard JK, Stephens M, Donnelly P. 2000. Inference of population structure using multilocus genotype data. Genetics155: 945–959.

7. Evanno G, Regnaut S, Goudet J. 2005. Detecting the number of clusters of individuals using the software STRUCTURE: A simulation study. Mol Ecol. 14: 2611–2620.

8. Jombart T. 2008. adegenet: a R package for the multivariate analysis of genetic markers. Bioinformatics 24: 1403–1405.
