# Supplementary material for: Are 150 km of open sea enough? Gene flow and population differentiation in a bat-pollinated columnar cactus
Source: PLoS One. 2023 Jun 29;18(6):e0282932. doi: 10.1371/journal.pone.0282932 (PMC10309638; doi:10.1371/journal.pone.0282932)
Supplement: Attachment — Submitted filename: response to reviewers.docx [file pone.0282932.s015.docx]

Response to reviewers

We appreciate the comments and queries made by the reviewers. These significantly improved the manuscript. We attended to all suggestions. Two citations were removed and several paragraphs deemed unnecessary were deleted. As a consequence, the reference section was updated. We added text to clarify some sections as requested by the reviewers. In total, the reduction of unnecessary text and additions asked by reviewers left the manuscript almost the same size. All figures are original and do not need copyright permission for publication. Figure 6 is redrawn from public data and the authors are acknowledged.

We confirm this work has not been published elsewhere, nor is it currently under consideration for publication, nor do we have conflicts of interest to disclose.

Abstract:

Corrected species name in italics.

Introduction:

Line 46, We described explicitly the query by the reviewer on founder effects

Following the suggested abridging and focusing of the introduction led us to reduce the content by deleting the following paragraphs:

Lines 49-52. The repetitive paragraph was deleted, including citation number 5.

Lines 60-64. Unnecessary information. The paragraph and citation 13 were deleted.

In line 64 we deleted "In"

line 88 deleted citation 23 (Karig), deleted ", and reduced gene flow"

line 115. added "]."

Line 114: *Myrtillocactus cochal* authorities are correct as originally stated in the ms. The basionym was described by C.R. Orcutt in 1889 and later assigned to *Myrtillocactus* by Britton & Rose. https://tropicos.org/name/5100588

Lines 115-116. Deleted "Their presence is likely the result of vicariant events."

Lines 119-123. Rearrangement of the sentence. Deleted as suggested "that has a highly specialized pollinator mutualism by moths [32],"

Line 123. Made clear that *Pachycereus pringlei* does not have an extensive distribution on the mainland.

Line 128. Added suggested comments on the narrow endemics at the tip of the peninsula (from line 114 onward).

Material and Methods:

Line 138. Added a brief description of the distribution range.

Line 167. Answered to the reviewer's question "Did you find you also had to manually adjust the alignment?" is yes. Added at the beginning of paragraph "After removing some nucleotides in the initial and final portion of the sequences (~15 nucleotides), multiple..."

Line 222, 223, 239. changed values to scientific notation

Line 248 Query by reviewer "What spatial relationship did the occurrences have? Was it necessary to do any thinning or adjustment to match the climate scale?" Yes. We tried to attain a homogeneous cover of the distribution range and bioclimatic envelope.

Results:

Line 398. Query by reviewer "This seems like perhaps the model was overfit somewhat. Maybe because .70 is a pretty high cutoff for correlation? The use of 9 climate variables is a lot of predictors. A pretty complex model." Added a note of caution highlighting the value of the three top bioclimatic variables.

Line 411. As suggested by the reviewer, the explanation of refuges was moved to the discussion.

Line 428. Reduced and moved to discussion

Discussion:

Line 434. Added subheadings to the discussion: 1) The role of vicariance and long-distance dispersal, 2) Ecological niche modeling and evolution, 3) The role of bats in shaping the genetic structure, and 4) Genetic structure suggests gene flow across the Gulf of California

Line 472. We rearranged the paragraph and included the niche modeling discussion suggested by reviewers in line 411.

Line 515. The query by the reviewer is relevant. "I guess it doesn't seem necessary to state the direction of dispersal? It seems highly unlikely that *thurberi* would have originated on the peninsula. But I could be wrong." We added a sentence explaining the possible evolutionary forces acting upon these populations.

Line 517. Made clear that Baja Californian populations are likely vicariant after the extensive separation of the peninsula.

Line 547. Made clear the lack of data on bat flight and seed dispersal.

Line 559. Agree. We might attempt it for the cardón sahueso (*Pachycereus pringlei*), widespread species of the islands.

Line 569. As suggested by the reviewer, we added a short paragraph summarizing the main findings
