# Supplementary material for: Are 150 km of open sea enough? Gene flow and population differentiation in a bat-pollinated columnar cactus
Source: PLoS One. 2023 Jun 29;18(6):e0282932. doi: 10.1371/journal.pone.0282932 (PMC10309638; doi:10.1371/journal.pone.0282932)
Supplement: Attachment — Submitted filename: response to reviewers.docx [file pone.0282932.s016.docx]

Response to reviewers

We greatly appreciate the comments and queries made by the reviewers. These significantly improved the manuscript. We attended to all suggestions, making a special effort in reducing and focusing the introduction. All mentions to text line numbers are made in relation to the original file.

Three citations were removed, and several paragraphs deemed unnecessary were deleted. Therefore, the reference section was updated. We added text to clarify some sections as requested by the reviewers. In total, the reduction of unnecessary text and additions asked by reviewers left the manuscript almost the same size. The text was thoroughly checked and small corrections of spelling and grammar were done. Only the major changes and responses to reviewers are detailed in the list line-by-line below.

All figures are original and do not need copyright permission for publication. Figure 6 is redrawn from public data and the authors are acknowledged. We confirm this work has not been published elsewhere, nor is it currently under consideration for publication, nor do we have conflicts of interest to disclose.

Abstract

Corrected species name in italics.

Introduction

Line 44, deleted “eventually”.

Lines 45-46, deleted " associated long-distance dispersal events and”.

Line 46, We described explicitly the query by the reviewer on founder effects. Added “when small number of colonizers result in reduced genetic variability.”

Following the suggested abridging and focusing of the introduction led us to reduce the content by deleting the following paragraphs: Lines 49-52. The repetitive paragraph was deleted, including citation number 5. Deleted “Examples of tectonics leading to the formation of land barriers include mountain development through fault blocks and alleviated valleys of the Basin and Range Province, reverse thrust, and the broad consequences of rifting of the continental crust [5].”

Line 53, deleted “the Pleistocene”.

Line 64, deleted "In" and corrected italics on “sky islands”.

Lines 66-67, deleted” For lower elevation taxa, dispersal better explains species distributions.”

Line 76, delete “random events like”, and “or”.

Line 81, as indicated by the reviewer, we added citations to support the peninsular effect.

Line 84 deleted “resulted in”, replaced it by “caused”.

line 88 deleted citation 23 (Karig), deleted ", and reduced gene flow". Corrected syntax attending reviewers suggestion.

Line 92, added “terrestrial” to make clear we are not talking about marine species.

Line 110, AS indicated by the reviewer, we corrected the species authorities.

Line 114: *Myrtillocactus cochal* authorities are correct as originally stated in the ms. The basionym was described by C.R. Orcutt in 1889 and later assigned to *Myrtillocactus* by Britton & Rose. <https://tropicos.org/name/5100588>

Lines 114-116. Deleted "Their presence is likely the result of vicariant events." The reviewer is right in indicating that the present distribution range could equally be the result of vicariance or geodispersal. Replaced with “and some narrow endemics providing further evidence of speciation by geographic isolation in peninsular conditions. The later include the little studied *Stenocereus eruca* (Brandegee) A.C. Gibson & K.E. Horak, *Lophocereus gatesii* M.E.Jones, and *Stenocereus littoralis* (K.Brandegee) L.W.Lenz.”

Line 115. added "]" next to the last parenthesis.

Lines 119-123. Rearrangement of the sentence. Deleted as suggested by the reviewer "that has a highly specialized pollinator mutualism by moths [32],".

Line 123. Attending the reviewer query, we made clear that *Pachycereus pringlei* does not have an extensive distribution on the mainland. Still, its mainland distribution range covers more than 400 km of coastal mainland and penetrates more than 100 km inland in the northern reaches of distribution.

Line 128. Added suggested comments on the narrow endemics at the tip of the peninsula (from line 114 onward).

Line 129, deleted “(2016).”

Material and Methods

Line 140. Added a brief description of the present distribution range, “Its extant distribution covers most subdivisions of the Sonoran Desert below 1000 m elevation, including northern Sinaloa, most of the state of Sonora in Mexico, scattered localities across southern Arizona in the USA, the lower half of the peninsula of Baja California, and some midriff islands of the Gulf of California (Figure 1).”

Lines 151-onward, added suggested quantitative description, “Using a clean knife rinsed in alcohol, a thin strip (8x1.5x0.2 cm) of green, heathy tissue was cut from the stem of 20 to 25 adult individuals under permit SEMARNAT 08-017-A. Specimens were arbitrarily selected across a site of at least one ha on each of the 12 sampled”

Line 167. Answered to the reviewer's question "Did you find you also had to manually adjust the alignment?" is yes. Added at the beginning of paragraph "After removing some nucleotides in the initial and final portion of the sequences (~15 nucleotides), multiple..."

Line 222, 223, 239. changed values to scientific notation.

Line 248 Query by reviewer "What spatial relationship did the occurrences have? Was it necessary to do any thinning or adjustment to match the climate scale?" Yes. We tried to attain as homogeneous cover of the distribution range and bioclimatic envelope as possible. Repeated collections were thinned. Even though, as in most surveys, most collections are arranged along major roads.

Results:

Line 398. Query by reviewer "This seems like perhaps the model was overfit somewhat. Maybe because .70 is a pretty high cutoff for correlation? The use of 9 climate variables is a lot of predictors. A pretty complex model." Added a note of caution about overfitting, highlighting the value of the three top bioclimatic variables.

Lines 405-407 were rewritten to

Line 411. As suggested by the reviewer, the explanation of LGM refuges was moved to the discussion.

Lines 425-onward. Reduced and moved to discussion.

Discussion:

Line 434. Added subheadings to the discussion: 1) The role of vicariance and long-distance dispersal, 2) Ecological niche modeling and evolution, 3) The role of bats in shaping the genetic structure, and 4) Genetic structure suggests gene flow across the Gulf of California.

Line 466, added section on Niche. Added sections moved from the results section “Niche models show that the climatically predicted habitat for the species matched the current geographic distribution of the species (Fig 5A; [26, 35]), suggesting that suitable habitat available to the species was substantially reduced during the LGM. Therefore, refugia were likely located in coastal areas of the southwestern Sonoran Desert. This location may be explained by the lower sea levels, maritime climate, and milder temperatures in southern coastal locations where the Las Guásimas, Zacate Blanco, El Palmar, Nopolo, and Balandra populations are found today (Fig 5B). Over time, increasing suitable habitat, inland and northwards, led to the present distribution range. Thus, it is very likely that populations showing high genetic diversities such as Zacate Blanco and Nopolo retain the ancestral haplotypes, as do the probable Pleistocene refuges found by Sanderson et al. [76] for saguaro.”

Line 472. We rearranged the paragraph and included the niche modeling discussion suggested by reviewers in line 411 and line 425 onwards.

Line 474, attending reviewers query added “The wide environmental differentiation across the peninsula suggests local adaptation to different water regimes.”

Line 496, The query by the reviewer about the southern peninsula being geologicall or climatically isolated is highly relevant. It is both. Added a short phrase making it explicit.

Line 498, Added subheading “The role of bats in shaping the genetic structure“

Line 515. The queries by the reviewer are relevant. 1) “guess it seems important to me to specify the difference between recent colonization and barriers to dispersal in this case. “, 2) "I guess it doesn't seem necessary to state the direction of dispersal? It seems highly unlikely that *thurberi* would have originated on the peninsula. But I could be wrong." Answers: To clarify these relevant questions we added a sentence explaining the possible evolutionary forces acting upon these populations, “(Sonoyta), a marginal population in the northernmost, driest portion of the range, has only haplotype (H10), suggesting ancient colonization and founder effects, geographic isolation, or ecological constraints leading to local adaptation and differentiation[89, 90]. Baja Californian populations are probably vicariant. They…”.

Line 517. Made clear that Baja Californian populations are likely vicariant after the extensive separation of the peninsula.

Line 522, corrected the sentence to state that gene flow can stop divergence. Added “That number of dynamically active migrants is theoretically sufficient to prevent genetic drift leading to population divergence [9].”

Line 524, Added subheading: “Genetic structure suggests gene flow across the Gulf of California”

Line 520-533. Sentence rewritten.

Line 547. Made clear the lack of data on bat flight and seed dispersal.

Line 559. Agree. We might attempt it for the cardón sahueso (*Pachycereus pringlei*), a widespread species of the islands with intriguing clues to the possible migratory routes of seed dispersal agents..

Line 569. As suggested by the reviewer, we added a short paragraph summarizing the main findings. “The divergence between the peninsular ancestral population occurred approximately 1.56 million years and Pleistocene refugia were present in both population groups. In *S. thurberi*, two phylogeographic histories occur, one in the mainland where extensive gene flow generates greater homogeneity between populations in the form of a network, and another in the peninsula where isolation by distance favors greater genetic divergence. Still, evidence of gene flow between the mainland and the peninsula is compelling.“
